# Black queen cell virus detected in Canadian mosquitoes

**DOI:** 10.1093/jisesa/iead016

**Published:** 2023-04-02

**Authors:** Cole Baril, Christophe M R LeMoine, Bryan J Cassone

**Affiliations:** Department of Biology, Brandon University, Brandon, Manitoba R7A 6A9, Canada; Department of Biology, Brandon University, Brandon, Manitoba R7A 6A9, Canada; Department of Biology, Brandon University, Brandon, Manitoba R7A 6A9, Canada

**Keywords:** apiary, Aedes, nectar, Picornavirales, transmission

## Abstract

Black queen cell virus (BQCV) is a ubiquitous honeybee virus and a significant pathogen to queen bee (*Apis mellifera*) larvae. However, many aspects of the virus remain poorly understood, including the transmission dynamics. In this study, we used next-generation sequencing to identify BQCV in *Aedes vexans* (*n* = 4,000) collected in 2019 and 2020 from Manitoba, Canada. We assembled de novo the nearly complete (>96%) genome sequence of the virus, which is the first available from North America and the first report of BQCV being harbored by mosquitoes. Phylogenetic tree reconstructions indicated that the genome had 95.5% sequence similarity to a BQCV isolate from Sweden. Sequences of a potential vector (*Varroa destructor*) and a microsporidian associated with BQCV (*Nosema apis*) were not identified in the mosquito samples, however, we did detect sequences of plant origin. We, therefore, hypothesize that the virus was indirectly acquired by mosquitoes foraging at the same nectar sources as honeybees.

Highly valued as pollinators, honeybees (*Apis mellifera* L.) can be infected by a myriad of potentially detrimental viruses (Allen and Ball 1996, McMenamin and Genersch 2015; Grozinger and Flenniken 2019). Since it was first reported in 1955, Black queen cell virus (BQCV) is one of the most common and widespread honeybee viruses (Tentcheva et al. 2004, Ellis and Munn 2005, Mondet et al. 2014). Adult bees infected with the virus remain largely asymptomatic, however, BQCV can induce considerable mortality in the developing queen bee larvae, causing their necrotic carcasses to blacken pupal cells (Spurny et al. 2017). Despite its importance and prevalence, BQCV remains among the least understood honeybee viruses. 

BQCV is classified as a Triatovirus, within the Dicistroviridae family and the order Picornavirales. The viral genome is composed of linear single-stranded, positive sense RNA of ~8,550 nucleotides in length (Kubaa et al. 2020). This includes two open reading frames (ORFs) encoding polyproteins containing non-structural (ORF1) and structural (ORF2) subunits (Spurny et al. 2017). The viral genome sequence is currently available from several geographical locations, including Europe (Tapaszti et al. 2009, Spurny et al. 2017, Kubaa et al. 2020), Asia (Li et al. 2023), Africa (Leat et al. 2000), and Australia (unpublished). However, to the best of our knowledge, no BQCV genome sequence has been reported from the Americas.

The precise mode of BQCV transmission has not yet been fully determined, but it likely involves multiple routes. Indeed, there is evidence that the virus is both venereally (Prodělalová et al. 2019) and vertically (Chen et al. 2006b, Naggar and Paxton 2020) transmitted. The presence of the microsporidian *Nosema apis* has also been linked to BQCV (Leat et al. 2000), but its role (if any) in virus transmission is unclear. Further, *Varroa* infestations have been associated with BQCV and the virus has been isolated from these mites (Ribière et al. 2008). However, the capacity of mites to serve as vectors of BQCV remains unresolved.

Another potential mode of BQCV transmission is through the foraging excursions of adult bees (Spurny et al. 2017). Honeybees collect nectar from flowers and orally pass it between workers, gradually converting the nectar into honey through biochemical processes and moisture loss (Leach and Drummond 2018). Although there is no direct evidence for nectar transmission of BQCV, honeybees have been shown to deposit the virus on flowers (Alger et al. 2019) and BQCV-positive pollen and honey have been identified (Chen et al. 2006a). Further, BQCV has been identified in both bumblebees (Peng et al. 2011) and solitary bees (Murray et al. 2018), which may be attributed to interspecies transfer of the virus through contaminated nectar sources.

Several methods have been employed to detect honeybee viruses, including enzyme-linked immunosorbent assay (ELISA), immunodiffusion, reverse transcriptase PCR (RT-PCR), and enhanced chemiluminescent (ECL) immunoblotting (Stoltz et al. 1995, Allen and Ball 1996, Benjeddou et al. 2001, Milićević et al. 2018). Next-generation sequencing (NGS) technologies are a relatively recent development and provide superior resolution and sensitivity to the aforementioned approaches. It has become increasingly used to detect viruses in insects, including honeybees (Marzoli et al. 2021, Li et al. 2022). In this investigation, we provide molecular evidence via NGS of *Aedes vexans* mosquitoes from Manitoba, Canada, harboring BQCV. We further speculate that the virus originated in flowers foraged by nectar-feeding mosquitoes.

## Materials and Methods

To conduct this research, we collected mosquitoes in 2019 and 2020 in conjunction with provincial (Manitoba) surveillance programs. CDC miniature light traps (Model 1012, John W. Hock, Gainesville, FL) were hung on trees ~1.5 m off the ground, which released carbon dioxide (CO_2_; a female mosquito attractant) at 15 pounds per square inch (PSI) from dusk until dawn. In 2019, we placed traps in ten locations across the city of Brandon (49°50ʹ54″N 099°57ʹ00″W) in coordination with the City of Brandon and Manitoba Public Health. Trapping took place two nights per week for ten weeks, from July to September. In 2020, one-time (July) satellite traps from nine additional locations throughout the central and eastern regions of the province were provided to us by the City of Winnipeg Insect Control Branch. The collected mosquitoes were sorted and *Ae. vexans* were identified using relevant mosquito identification keys (Wood et al. 1979, Thielman and Hunter 2007). It should be noted that only females were captured and sequenced, as CO_2_ does not elicit host-seeking behaviors in males. Mosquitoes were stored at −80°C in location- and date-specific Petri dishes.

A maximum of fifty mosquitoes were pooled and RNA was isolated using the RNeasy Mini Kit (Qiagen, Hilden, Germany) according to the manufacturer’s recommendations. We then combined the RNA into two pools (by year) and ~2 µg per sample was sent to the Génome Québec Innovation Centre (McGill University, Montreal, QC, Canada) for mRNA library preparation (New England Biolabs, Ipswich, MA, USA) and paired-read sequencing (100 bp) using the NovaSeq 6000 System (Illumina, San Diego, CA). A total of 1,783 and 2,208 pooled *Ae. vexans* individuals were sequenced from collections in 2019 and 2020, respectively. Raw RNA sequencing reads can be retrieved from the NCBI short sequence read archive under the SRA accession number PRJNA866544. We processed the raw reads and performed *de novo* contig assembly using the CLC Genomics Workbench version 20 and optimized parameters: mismatch cost = 2, insertion cost = 3, deletion cost = 3, length fraction = 0.7, and similarity fraction = 0.95. To identify BQCV, each contig was mapped against the NCBI non-redundant (nr) database using BLASTn (E-value < 1 × 10^100^). We confirmed the presence of BQCV and the viral genome assembly using default settings with the Chan Zuckerberg ID Metagenomic Pipeline v6.8 (Chan Zuckerberg Biohub; CZID), an open-sourced cloud-based bioinformatics platform (Kalantar et al. 2020).

To explore the evolutionary relationship among BQCV isolates, we constructed a maximum likelihood tree based on a 7,961 bp region of the genome (>93% of the complete sequence). In addition to our Canadian strain, BQCV genomic sequences were retrieved from the NCBI database to represent a breadth of geographical viral isolates. We retained only genomic sequences that were >99% complete, and when more than one isolate sequence was available we selected the two most divergent isolates for further analysis. The sequences were aligned using default parameters (pairwise gap opening penalty = 15, gap extension penalty = 6.66) and tested for the best evolutionary model in MEGA X (Kumar et al. 2018). The tree with the highest likelihood was selected (-35842.56) and the phylogeny was then inferred using a maximum likelihood approach and a General Time Reversible (GTR) model with discrete Gamma distribution (5 categories; +G, parameter = 0.1639), with 1,000 bootstrap iterations (Nei and Kumar 2000). All codon positions were included in the analysis, while gaps and missing data were discarded from the final analysis.

## Results

**Fig. 1.**
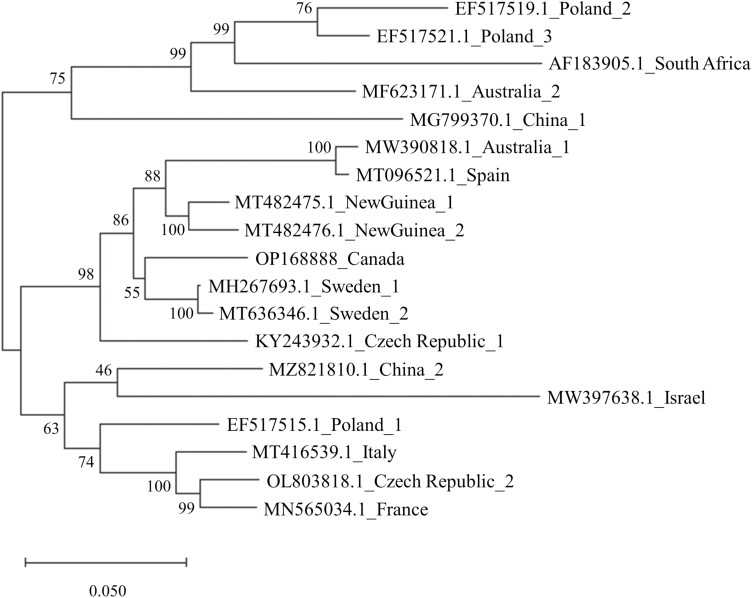
Maximum likelihood inference of evolutionary relationship amongst BQCV isolates worldwide. BQCV sequences were retrieved and aligned to the Canadian isolate (OP168888) genomic sequences in MEGA X (see methods). The tree is drawn to scale with branch lengths representing the average number of substitutions per site analyzed. Numbers near branches represent the percentage of trees supporting the proposed topology between isolates.

**Fig. 2.**
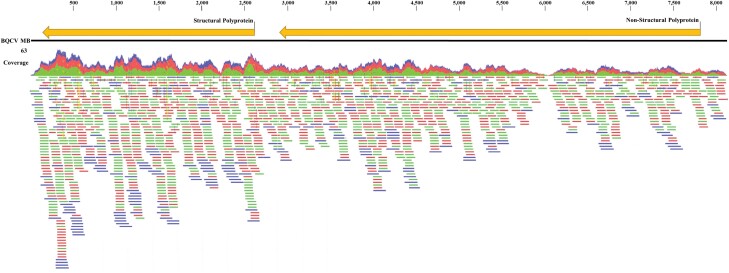
Coverage map of the BQCV genome of the Canadian isolate. The map was generated by mapping sequencing reads from both positive *Aedes vexans* samples to the most similar BQCV genome in NCBI (MH267693.1). The mapping was carried out using CLC and default settings.

RNA sequencing of two pooled *Ae. vexans* RNA samples (2019 and 2020) generated 91,331,949 raw sequence reads. The reads were assembled into 28,566 (2019) and 33,563 (2020) contigs which, as expected, were primarily of *Aedes* origin. In 2019, one contig matched to BQCV, which had 95.5% sequence similarity to a BQCV isolate from Sweden in NCBI (MH267693.1; Fig. 1). The sequence represented ~95% (8,122 bp) of the viral genome with ~329 bp of the 5ʹ region not present, presumably due to low coverage of that region. For 2020, seven smaller BQCV contigs (between 267 and 826 bp) were assembled representing ~38% (3,233 bp) of the genome. We constructed a coverage map of the BQCV isolate from Canada using sequencing reads from both 2019 and 2020, which are displayed in Fig. 2. Genomic features of the BQCV sequence (e.g., ORFs, predicted proteins) were consistent with those previously reported (Leat et al. 2000, Spurny et al. 2017, Kubaa et al. 2020). The genomic sequence of the BQCV isolates reported in this study has been deposited in the GenBank database under the accession number OP168888.

To provide insights into the possible origins of BQCV in *Ae. vexans*, we searched each contig sequence for orthologue matches that may be derived from potential vectors (mites), nectar (plants), and a microsporidian associated with the presence of BQCV in infected honey bee larvae (*Nosema apis*). For both samples, no contigs were identified that mapped to the most recent genome assemblies of *Varroa destructor* (Vdes_3.0) or *Nosema apis* (NapisBRLv01). However, a subset of contigs from the 2020 collections (*n* = 3) was of chloroplast origin (Table 1). All three are contigs mapped to multiple species with identical coverage/sequence similarity, including flowering plants, trees, and shrubs.

**Table 1.** Contig sequences of plant origin found within *Aedes vexans* samples that were positive for BQCV

Contig size (bp)	Year collected	Top match	Query coverage	Sequence similarity	Sequence
399	2020	Chloroplast	100%	100%	CGAATAGGTCAACCTTTCGAACTGCTGCTGAATCCATGGGCAGGCAAGAGACAACCTGGCGAACTGAAACATCTTAGTAGCCAGAGGAAAAGAAAGCAAAAGCGATTCCCGTAGTAGCGGCGAGCGAAATGGGAGCAGCCTAAACCGCGAAAACGGGGTTGTGGGAGAGCAATACAAGCGTCGTGCTGCTAGGCGAAGCGGTGGAGTGCTGCACCCTAGATGGCGAGAGTCCAGTAGCCGAAAGTATCACTAGCTTACGCTCTGACCCGAGTAGCATGGGACACGTGGAATCCCGTGTGAATCAGCAAGGACCACCTTGCAAGGCTAAATACTCCTGGGTGACCGATAGTGAAGTAGTACCGTGAGGGAAGGGTGAAAAGAACCCCCATCGGGGAGTGA
473	2020	Chloroplast	100%	99.15%	CTACCTTAGGACCGTTATTGTTACGGCCGCCGTTCACCGGGGCTTCGGTCGCCGGCTCCCCAGTCATCAGGTCACCAACATCCTTGACCTTCCGGCACTGGGCAGGCGTCAGCCCCCATACATGGTCTTACGACTTTGCGGAGACCTGTGTTTTTGGTAAACAGTCGCCCGGGCCTGGTCACTGCGACCCCCTTTGTGAGGAGGCACCCCTTCTCCCGAAGTTACGGGGCTATTTTGCCGAGTTCCTTAGAGAGAGTTGTCTCGCGCCCCTAGGTATTCTCTACCTACCCACCTGTGTCGGTTTCGGGTACAGGTACCCTTTTGTTGAAGGTCGTTCGAGCTTTTCCTGGGAGTATGGCATCGGTTACTTCAGCGCCGTAGCGCCTTGGTACTCGAACATTGGCTCGAGGCATTTTCTCGACCCCTTCTTACCCTGAAAAAGCAGGGGCACCTTGCGTCCTTGAACCGATAAC
226	2020	Chloroplast	100%	100%	GCTAAGCGATCTGCCGAAGCTGTGGGATGTAAAAATGCATCGGTAGGGGAGCGTTCCGCCTAGAGGGAAGCACCCGCGCGAGCAGTGGTAGACGAAGCGGAAGCGAGAATGTCGGCTTGAGTAACGCAAACATTGGTGAGAATCCAATGCCCCGAAAACCTAAGGGTTCCTCTGCAAGGTTCGTCCACGGAGGGTGAGTCAGGGCCTAAGATCAGGCCGAAAGGCG

All three sequences could not be resolved to the species level, with each having top matches to flowering plants, shrubs, and trees.

## Discussion

The results of our study strongly suggest the presence of BQCV in *Ae. vexans* is due to nectar foraging behaviors. This may seem counterintuitive as females of this mosquito species are hematophagous, primarily feeding on the blood of large mammals (e.g., deer, horses, and cows) (Nasci 1984). The nutrients in vertebrate blood are required for egg production by the vast majority of mosquito species, including iron and amino acids (Goldstrohm et al. 2003, Zhou et al. 2007). However, nectar represents a key source of nutrition for adult mosquitoes of both sexes (Barredo and DeGennaro 2020). For females, sugar deprivation has been associated with both reduced survival and fecundity (Foster 1995, Fernandes and Briegel 2005, Chadee et al. 2014). Since all of the plant sequences identified in our *Ae. vexans* samples were derived from chloroplasts, which are highly conserved (Cheng et al. 2020), we were unable to resolve the nectar sources to the species level. Plant preferences differ by mosquito species and availability, with a variety of semiochemicals elicited by plants that serve as attractants (Barredo and DeGennaro 2020). There is currently no evidence that BQCV can replicate in mosquitoes or be transmitted by mosquitoes, indicating *Ae. vexans* is likely a dead-end host for the virus. However, our findings of the virus in the same mosquito species across multiple years suggest that BQCV may be commonly associated with nectar foraging *Ae. vexans*.

To our knowledge, this is the first report of BQCV detected in mosquitoes or any other dipteran. In addition to mites, the virus has been identified in several Hymenopterans including ants (Payne et al. 2020), bumblebees (Peng et al. 2011), solitary bees (Murray et al. 2018), and wasps (Singh et al. 2010). Interspecies transmission of BQCV has been hypothesized to be due to direct (e.g., parasitism, predation, and scavenging) and/or indirect (foraging at the same nectar sources) interactions between honeybees and these arthropods (Grozinger and Flenniken 2019, Payne et al. 2020). Future studies aimed at directly sampling nectar-feeding *Ae. vexans* and validating the presence of BQCV in both the nectar and mosquito would better implicate foraging behaviors as a direct source of this virus in mosquitoes.

Our study also highlights the capabilities of massive parallel NGS technologies to characterize aspects of the host microbiome. Although it requires considerable integration of bioinformatics (Conesa et al. 2016), many limitations of traditional approaches for pathogen identification (e.g., PCR-based methods and serological testing) can be overcome using NGS. In addition to its greater resolution and sensitivity, NGS does not require a priori knowledge of the nucleic acid to be sequenced or specific antibodies (Díaz Cruz et al. 2019). Indeed, we had no expectation of identifying BQCV in *Ae. vexans* or isolating the near complete viral genome sequence. It is conceivable that NGS could be harnessed to determine the prevalence of key pollinator pathogens (e.g., Deformed wing virus, Acute bee paralysis virus, Kashmir bee virus, and Sacbrood virus) in a given region, either by sequencing the animal or the nectar. Moreover, if the virus can be readily obtained from nectar, it could serve as a biomarker to determine pollinator plant preferences and/or potential outbreaks.

In conclusion, we present the first report of BQCV detected in mosquitoes as well as the first comprehensive genome sequence of the virus from North America. While other studies have detected the virus in arthropods using traditional approaches (e.g., PCR), we demonstrate that BQCV can also be readily detected using NGS technologies. We hypothesize that *Ae. vexans* acquired BQCV by foraging at the same nectar sources as honeybees harboring the virus. Future research is needed to investigate whether mosquitoes are capable of transmitting BQCV to honeybees, either directly or indirectly. Further, studies aimed at determining the pervasiveness of BQCV in mosquito species and the contaminated nectar sources would provide added insights into the transmission dynamics of this virus.
